# B7-H3, a checkpoint molecule, as a target for cancer immunotherapy

**DOI:** 10.7150/ijbs.41105

**Published:** 2020-03-25

**Authors:** Shuo Yang, Wei Wei, Qi Zhao

**Affiliations:** 1 Institute of Translational Medicine, Faculty of Health Sciences, University of Macau, Taipa, Macau SPR, China.; 2 Cancer Centre, Faculty of Health Sciences, University of Macau, Taipa, Macau SPR, China.; 3 Biological Imaging & Stem Cell Core, Faculty of Health Sciences, University of Macau, Taipa, Macau SPR, China.; 4 Guangdong Cord Blood Bank; Guangzhou Municipality Tianhe Nuoya Bio-engineering Co. Ltd, Guangzhou, China

**Keywords:** B7-H3, CD276, cancer immunotherapy, antibody, immune checkpoint

## Abstract

B7-H3 (also known as CD276) is a newly found molecule of B7 family, which may be a promising target for cancer treatment. B7-H3 protein was demonstrated to be expressed in several kinds of tumor tissues including non-small-cell lung cancer (NSCLC) and prostate cancer. Its expression is highly associated with undesirable treatment outcomes and survival time, due to function of the immune checkpoint molecule. It was classified as either a co-stimulatory molecule for T cell activation or the nonimmunological role of regulating signaling pathways. Although there is still no agreed conclusion on the function of B7-H3, it may be a valuable target for cancer therapy. This review aims to provide a comprehensive, up-to-date summary of the advances in B7-H3 targeting approaches in cancer therapy. Although several challenges remain, B7-H3 offers a new therapeutic target with increased efficacy and less toxicity in future cancer treatment.

## Introduction

**Figure 1:**
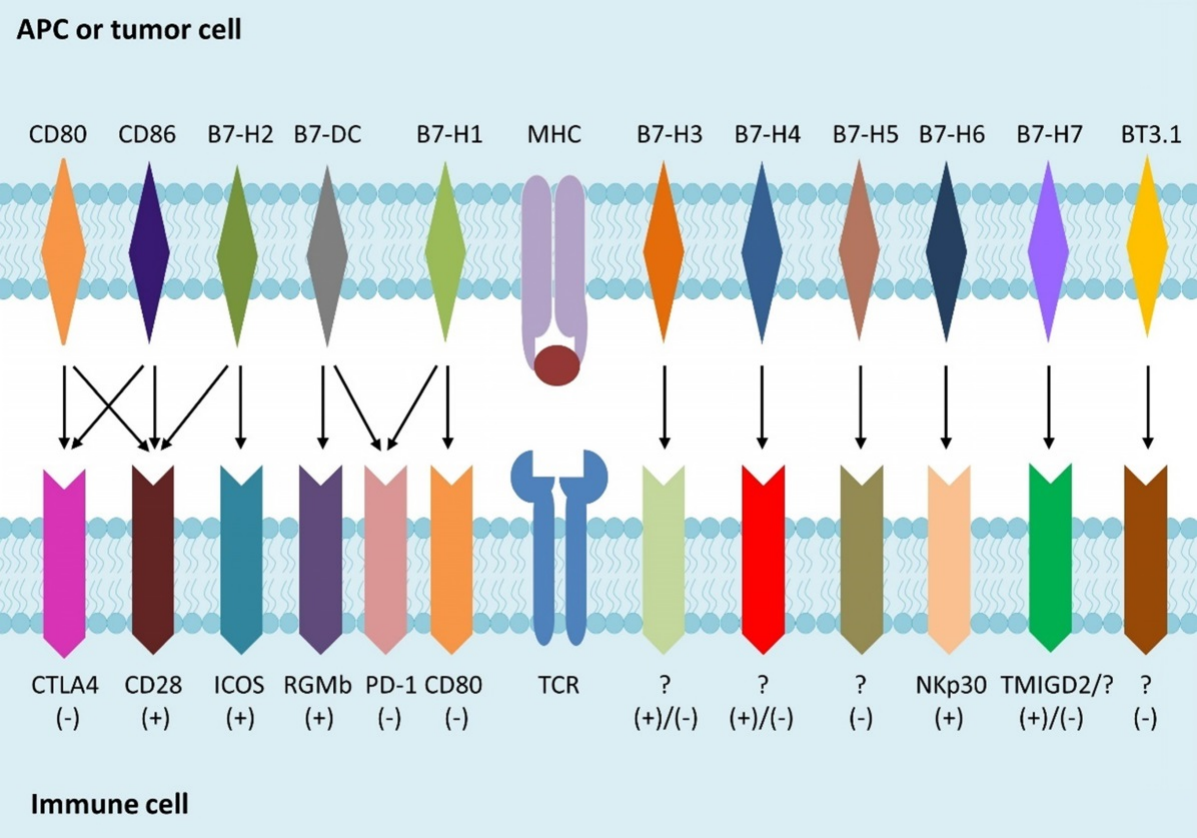
** B7 family members and their receptors.** B7 is a type of peripheral membrane protein found on activated APC. A specific antigen is presented by MHC molecules on APCs or tumor cells to TCR on T cells, to form the first signal for the activation of T cells. B7 family members as well as other co-stimulatory molecules binding to their receptors expressed on T cells, which is called the second signal, is used to direct and/or fine-tune the responses of T cells.

Immunotherapy is a newly individualized treatment that activates or suppresses the immune system to amplify or diminish an immune response and has been developed rapidly for treating various forms of cancer in recent years. Immunotherapy for cancer, such as chimeric antigen receptor (CAR)-T cells, CAR-natural killer (NK) cells, PD-1 and PD-L1 inhibitor, aims to help patients' immune system fight cancer 1. The activation of T cell depends on both the specific combination of T cell receptor (TCR) and peptide-bound major histocompatibility complex (MHC), and the interplay of co-stimulatory molecules of T cell with ligands on antigen presenting cells (APCs). The B7 families, peripheral membrane proteins on activated APCs, have been shown to participate in regulation of T cell responses (Fig. 1). Recent studies indicated that the up-regulation of inhibitory B7 molecules in the cancer microenvironment was highly related to the immune evasion of tumor 2. As a newly identified member of the B7 family 3, B7-H3 could promote the activation of T cells and the proliferation of IFN-γ. In this review, we summarized research in recent years, focusing on the functional properties of B7-H3 and its potential role in the recent progress of cancer immunotherapy. We will also discuss the benefits, challenges, and considerations of targeting B7-H3 therapy in clinical development.

## B7 family

Different B7 molecules have either positive or negative co-stimulatory signals while modulating immune cell responses (Table 1) 4, 5. Immune checkpoints, such as PD-1, PD-L1, PD-L2, and CTLA4, are molecules holding many receptor-ligand interactions to evade the immune system and facilitate proliferation. Several monoclonal antibodies (mAbs) that block these proteins were developed to down-regulate the inhibitory immune response, and promote the cellular cytotoxicity of T cells that eliminate tumor cells 6. Among the immune checkpoint-blocking drugs, the inhibitors targeting PD-1 or CTLA4 were successfully used for treating patients with metastatic melanoma, with improved responses and prolonged survival 7. This success led to the development of such agents for treating a wide range of malignancies, including renal cell carcinoma (RCC) 8, NSCLC 9-11, and acute myeloid leukemia (AML) 12, which further enhanced the response rate compared to conventional treatments, and prolonged the survival time of patients.

## Biological function of B7-H3

B7-H3 was found to be overexpressed among several kinds of human cancer cells and was correlated with disease deteriorations 13-19 (Table 2). B7-H3 was recognized as a co-stimulatory molecule for immune reactions such as T cell activation and IFN-γ production 20. In the presence of anti-CD3 antibody mimicking the TCR signal, human B7-H3-Ig fusion protein was found to increase the proliferation of both CD4^+^ and CD8^+^ T cells and enhance the cytotoxic T lymphocyte (CTL) activity *in vitro*. An orthotopic colon cancer model of mice was constructed by researchers who studied the mechanism of B7-H3 antitumor ability 21. Their data suggested there was an antitumor effect of B7-H3 on adenocarcinoma of the colon, which could also be regarded as a promising therapy for the treatment of colon cancers. Moreover, they detected the co-stimulatory molecule role of B7-H3 in the model of colon cancers established by orthotopic injection 22. In a study among human pancreatic cancer patients, B7-H3 was recognized as a co-stimulatory molecule that was not only abundantly expressed in pancreatic cancer but also associated with increased treatment efficacy 23. They found that although B7-H3 expression was detectable in most examined pancreatic cancer samples, and significantly upregulated in pancreatic cancer versus normal pancreas, patients with high tumor B7-H3 levels had a significantly better postoperative prognosis than patients with low tumor B7-H3 levels.

Recently, the inhibitory ability of B7-H3 towards T cell proliferation was discovered. The proliferation of both CD4^+^ and CD8^+^ T cells could be inhibited by B7-H3 24. Moreover, in synovial monocytes, the expression of surface B7-H3 was found to correlate inversely with the rheumatoid arthritis (RA) clinical parameters 25. Another study 26 indicated that in oral squamous cell carcinoma (OSCC), larger tumor size, advanced clinical stage, and low survival rate of patients were positively associated with B7-H3 overexpression. In addition, tumor cell proliferation was suppressed when B7-H3 was blocked, and tumor growth was enhanced when B7-H3 expression was restored.

B7-H3 remains an orphan ligand, although a potential receptor, TLT-2, was detected on activated immune cells 27. B7-H3/TLT-2 was shown to augment chemokine production and proinflammatory cytokine, by activating the phosphorylation of downstream mitogen-activated protein kinase (MAPK) p38 and NF-kappa B p65 28. However, similar effects were not seen in another study of both human and murine B7-H3 29. Thus, there is not enough evidence for TLT-2 as a receptor of B7-H3, and specific receptors capable of binding B7-H3 need to be conclusively identified. This may account for the contradictory co-stimulatory and co-inhibitory roles that B7-H3 plays in immune response.

## Roles of B7-H3 in tumor progression and drug resistance

Mechanisms for the correlation between B7-H3 and tumor progression were deeply explored from immunological and non-immunological aspects. For NSCLC patients, B7-H3 and regulatory T cells (Tregs) were identified as having potential cooperative role in the immune evasion of tumor cells, and the resulting poor outcomes 30. B7-H3 and CD14 were shown to be co-expressed in RCC tissues, which were positively associated with tumor progression, indicating that the important role B7-H3 played in angiogenesis of RCC might be influenced by CD14^+^ monocytes 31. Similarly, co-expression of B7-H3 and CD133 was evidently associated with progression of CD133^+^ colorectal cancer 32. In colorectal carcinoma (CRC) tissues, expression of B7-H3 and infiltrating macrophage density were found to be positively associated, while both were negatively correlated with patients' survival rate. Since the presumed receptor of B7-H3 was found on activated monocytes and macrophages, these results further indicated the potential role of B7-H3 signal and macrophages in tumor progression 13. High B7-H3 expression was found in human breast cancer tissues and may play an important role in tumor progression and invasiveness. This expression appeared to increase the ability of B7-H3 to promote secretion of the immunosuppressive cytokine IL-10 33. Another inhibitory molecule, immunoglobulin-like transcript 4 (ILT4), was demonstrated to increase B7-H3 expression through PI3K/AKT/mTOR signaling, which reduced T infiltrating lymphoid cells (TILs) and led to lower overall survival 34. B7-H3 was shown to influence tumor progression by regulating the relative molecules via JAK2/STAT3 pathway in several types of cancer 35-37. Moreover, expression of B7-H3 and tyrosine kinase receptor Tie-2 in clear cell renal cell carcinoma (ccRCC) tumor vasculature were closely related to the progression and prognosis of the disease, while ccRCC angiogenesis was possibly promoted by B7-H3 through the Tie-2 pathway 14. Furthermore, soluble B7-H3 (sB7-H3) was reported to promote the invasion and metastasis of pancreatic carcinoma cells through the TLR4/NF-κB pathway 38.

Emerging studies have also demonstrated that B7-H3 may contribute to the resistance of anti-cancer drugs with various mechanisms. Proliferation and glycolytic capacity of metastatic melanoma cells were found to decrease when expression of B7-H3 was reduced or inhibited, leading to reduced resistance to chemotherapy as well as other targeted therapies 39; this mechanism was further demonstrated to involve the inactivation of p38 MAPK signaling 40. Similarly, glycolytic capacity was increased with the overexpression of B7-H3 in tumor cells, which induced the resistance to API-2 (triciribidine) and everolimus (RAD-001) 41. In contrast, suppression of B7-H3 was found to improve the sensitivity of human breast cancer cell lines to chemotherapy agent paclitaxel, while overexpression of B7-H3 led to the resistance of cancer cells to the drug; this was identified as at least partially relative to interference with the Jak2/Stat3 pathway 42. Overexpression of B7-H3 could increase the population of cancer stem cell and induce cancer cell resistance to drugs by activating MEK through major vault protein (MVP) 43. B7-H3 can confer colorectal cancer cell resistance to 5-fluorouracil (5-FU) by increasing the expression of thymidylate synthase (TS) and activating PI3K/ Akt/TS signaling 44. In pancreatic carcinoma cells, B7-H3 was found to induce gemcitabine resistance, at least partially due to the downregulated survivin expression 45.

## Targeting B7-H3 therapy

A number of anti-B7-H3 approaches have been studied in preclinical or clinical trials. The details of several agents under clinical trials are described in this section and summarized in Table 3.

Enoblituzumab (MGA271), a humanized mAb targeting B7-H3, mediates potent antibody-dependent cellular cytotoxicity (ADCC) against a broad range of tumor types. For example, it was investigated in treating refractory B7-H3-expressing tumors such as melanoma (NCT01391143), and B7-H3-expressing neoplasms including osteosarcoma and Ewing's sarcoma (NCT02982941). Furthermore, MGA271 exhibited potent antitumor activity in xenograft models of B7-H3-expressing renal cell and bladder carcinoma. And in cynomolgus monkeys, no significant safety findings were discovered by toxicology studies 46.

Another mAb called 8H9, was derived from the fusion of mouse splenic lymphocytes and myeloma SP2/0 cells of BALB/c mice that were immunized by human neuroblastoma 47. Since B7-H3 is both an immune inhibitory ligand and an antigen in many solid tumors, researchers humanized and affinity- matured the anti-B7-H3 mouse mAb 8H9 based on *in silico* modeling and affinity maturation via yeast display 48. It was concluded that humanized 8H9 antibodies could regulate the inhibitory immune properties of B7-H3 on target tumors and affect the immune checkpoint blockade. Like other bispecific antibodies (Bi-Abs) that validated for treating various diseases 49, 50, activated T cell (ATC) armed with the anti-CD3 x anti-B7-H3 (B7-H3Bi-Ab), had specific cytotoxic activity against tumor cells by ADCC. Compared to unarmed ATC, enhanced cytotoxic activity and cytokine secretion of B7-H3Bi-armed ATC were observed. Infusion of B7-H3Bi-armed ATC also inhibited tumor growth *in vivo*, and significantly improved survival 51.

Researchers explored the antitumor ability of the antibody-drug conjugates (ADCs) that specifically destroyed B7-H3 positive expressing tumors, and found that established tumors and metastases were eradicated, and overall survival improved significantly, which demonstrate the anti-CD276-drug conjugates as promising reagents for highly selective broad-acting anti-cancer therapies 52. A promising exatecan derivative (DX-8951 derivative, DXd), used for drug conjugation as DXd-ADC targeting B7-H3, showed effective antitumor efficacy as well as less adverse effects 53. 131I-labeled anti-B7-H3 mAb (131I-4H7) had radiobiological and treatment effects on nude mice with human RCC. 131I-4H7 was markedly absorbed by RCC xenografted tumor, and the development of tumor was inhibited by 131I-4H7 significantly 54. In addition, constructed bioconjugates targeting both B7-H3 and chlorin e6 have been shown to have the ability of treating NSCLC both *in vitro* and *in vivo* under the guidance of spectroscopic photoacoustic and fluorescence imaging, and could display effective tumor diagnosis and therapy as a novel approach of immunotherapy 55.

Recently, CAR-T cells that were genetically engineered to graft specific recognition ability for T cells were generated with B7-H3 as the target 56. In this research, they explored the treatment efficacy of CAR-T cells targeting B7-H3 on pancreatic ductal adenocarcinoma, ovarian cancer, and neuroblastoma, both *in vitro* and in orthotopic and metastatic xenograft mouse models including patient-derived xenograft. They found that the growth of tumor could be controlled without evident toxicity. Antitumor effects of B7-H3-specific CAR-T cells were also assessed in primary glioblastoma cell lines. The specific antitumor functions of CAR-T cells were confirmed both *in vitro* and* in vivo* 57. There is rapid development of antibody drugs and CAR-T cells that target B7-H3, which may be administered alone or may achieve synergistic anti-tumor effects when combined with chemotherapeutic agents or other therapeutic regimens.

## Conclusion

Immunotherapy represents a new promising therapeutic approach for several cancers, and has the specific advantage of more efficacy, less side effects, and less complex processes compared to therapies such as surgery and chemotherapy. Recent research has provided strong evidence for the value of B7-H3 as a target in immune-based antitumor therapies, for its overexpression across several kinds of cancer cells but seldom in normal cells. Although B7-H3 was shown to exhibit inhibitory effects in modulating both T cells and NK cells, several studies found that B7‐H3 could regulate immune response towards target organs in a costimulatory manner. Until now, there is still no unified view on the receptor of the B7-H3 molecule. More research is needed to identify the mechanism of the two regulatory functions of B7-H3 and to detect its effective receptor, to further understand its regulation of immune response and develop valuable drug targets. In addition, nonimmunological roles of B7-H3 that associated with different proteins, may affect cancer migration, invasion, and angiogenesis by interacting with relative signaling pathways. Moreover, since B7-H3 was found broadly expressed by both tumor cells and tumor vasculature, and upregulated in clinical samples of human cancer metastases, it could be regarded as a potential marker for immune evasion of tumor cell.

Compared to other immune checkpoints, B7-H3 appears to be a unique and powerful target in cancer immunotherapy, as it not only influences innate and adaptive immunity but also regulates aggressiveness of cancer cells through various non-immunological pathways. Verification of the receptor for B7-H3 and better elucidation of B7-H3 pathway in immune response and cancer development is crucial and may help to provide rationale for therapeutic application of anti-B7-H3 agents in clinical patients. Further understanding of the role of B7-H3 and further preclinical and/or clinical exploration may establish this as a reasonable anti-tumor target and anti-metastatic marker.

## Figures and Tables

**Table 1:** Different roles of B7 family members in immune cell response.

Name	Alternative name	Role in immune cell response
B7-1	CD80	Activation/inhibition
B7-2	CD86	Activation/inhibition
B7-DC	PD-L2, CD273	Activation/inhibition
B7-H1	PD-L1, CD274	Inhibition
B7-H2	ICOSL	Activation
B7-H3	CD276	Activation/inhibition
B7-H4	B7S1, B7x, Vtcn1	Activation/inhibition
B7-H5	VISTA, GI24, Dies1, PD-1H	Inhibition
B7-H6	NCR3LG1	Activation
B7-H7	HHLA2	Activation/inhibition
BT3.1	BTF5, CD277, BTN3A1	Inhibition

**Table 2:** B7-H3 expression in multiple types of human cancers.

Cancer type	Case number	% Positive	Findings	Refs
Non-small lung cancer	82	74	B7-H3 expression was correlated with ineffective anti-PD-1 immunotherapy	15
Cutaneous squamous cell carcinoma	66	85	Tumor B7-H3 expression was higher in immunocompetent patients	16
Pancreatic cancer	26	65.4	No positive cells were detected in normal pancreas specimens	17
Primary hepatocellular carcinoma	70	88.57	Expression of B7-H3 promoted tumor progression	18
Colorectal carcinoma	117	96.6	B7-H3 expression was negatively associated with overall survival rate	13
Clear cell renal carcinoma	82	97.56	B7-H3 is associated with the tumor-node-metastasis stage of patients	14
Breast cancer	74	56.8	B7-H3 expression was mainly observed in cell membrane and cytoplasm	19

**Table 3:** Summary of anti-B7-H3 approaches in clinic.

Format	Drug	Tumor type	Developer	Highest trail stage
^a^ADC and ADCC	Enoblituzumab (MGA271)	Prostate cancer, melanoma, HNSCC, NSCLC, urothelial cancer, neuroblastoma, rhabdomyosarcoma, osteosarcoma, Ewing sarcoma, Wilms' tumor, DSRCT or malignant solid tumors of any other histology that test positive for B7-H3	MacroGenics	Phase 2
	^131^I-Omburtamab	CNS/leptomeningeal metastases, DSRCT and other solid tumors involving the peritoneum	Y-mAbs Therapeutics	Phase 3
	177Lu-DTPA-omburtamab	Medulloblastoma	Y-mAbs Therapeutics	Phase 2
	^131^I-8H9	Peritoneal cancer, neuroblastoma, CNS/leptomeningeal metastases	Y-mAbs Therapeutics	Phase 3
	^124^I-8H9	Brain cancer, brain stem glioma	Y-mAbs Therapeutics	Phase 1
	MGC018	solid tumors	MacroGenics	Phase 1
	DS-7300a	Advanced solid tumor malignant	Daiichi Sankyo	phase 2
Bispecific antibody	Orlotamab (MGD009)	Mesothelioma, bladder cancer, melanoma SCCHN, NSCLC, ccRCC, ovarian cancer, TNBC, pancreatic cancer, prostate cancer, colon cancer, soft tissue sarcoma	MacroGenics	Phase 1
CAR T-cell therapy	SCRI-CARB7H3	CNS tumor, DIPG, DMG, Ependymoma medulloblastoma, germ cell tumor, atypical teratoid/rhabdoid tumor, primitive neuroectodermal tumor, choroid plexus carcinoma pineoblastoma, glioma	Seattle Children's Hospital	Phase 1
Combination therapies	MGD009 in combination with MGA012	Advanced solid tumors	MacroGenics	Phase 1
	MGC018 with or without MGA012	Advanced solid tumors	MacroGenics	Phase 2
	MGA271 in combination with Pembrolizumab or MGA012	Melanoma, SCCHN, NSCLC, urethelial carcinoma	MacroGenics	Phase 1
	MGA271 in combination with Ipilimumab	Melanoma, NSCLC	MacroGenics	Phase 1
	B7-H3 CAR-T in combination with Temozolomide	Recurrent glioblastoma, refractory Glioblastoma	Second Affiliated Hospital, School of Medicine, Zhejiang University	Phase 2

^a^ADC and ADCC refer to antibody drug conjugate (ADC) therapies drugs and drugs target B7-H3 through antibody-dependent cell-mediated cytotoxicity (ADCC)

## Abbreviations

NSCLC: non-small-cell lung cancer
CAR: chimeric antigen receptor
NK: natural killer
TCR: T cell receptor
MHC: major histocompatibility complex
APC: antigen presenting cell
RCC: renal cell carcinoma
AML: acute myeloid leukemia
CTL: cytotoxic T lymphocyte
RA: rheumatoid arthritis
OSCC: oral squamous cell carcinoma
MAPK: mitogen-activated protein kinase
Treg: regulatory T cell
CRC: colorectal carcinoma
ILT: immunoglobulin-like transcript
ccRCC: clear cell renal cell carcinoma
sB7-H3: soluble B7-H3
MVP: major vault protein
5-FU: 5-fluorouracil
mAb: monoclonal antibody
ADCC: antibody-dependent cellular cytotoxicity
ATC: activated T cell
Bi-Ab: bispecific antibody
ADC: antibody-drug conjugate
